# Evaluation of differences in presentation and postoperative outcomes after adrenalectomy for pheochromocytoma according to age

**DOI:** 10.1530/EC-25-0604

**Published:** 2026-07-28

**Authors:** Sukhdeep Jatana, Kevin Verhoeff, Yanbo Wang, Zhicheng Wang, Jiale Zhou, Xiaorong Wu, Yonghui Chen, Maciej Śledziński, Andrzej Hellmann, Marco Raffaelli, Francesco Pennestrì, Mark S Sywak, Alexander Papachristos, Fausto F Palazzo, Tae Yon Sung, Byung-Chang Kim, Yu-Mi Lee, Fiona Eatock, Hannah Anderson, Maurizio Iacobone, Daryl K Gray, Richard C Chaulk, Vasilis Kosmoliaptsis, Nicola Colucci, Harry V M Spiers, Albertas Daukša, Özer Makay, Yiğit Türk, Hafize Basut Atalay, Els J M Nieveen van Dijkum, Anton F Engelsman, Isabelle Holscher, Gabriele Materazzi, Leonardo Rossi, Chiara Becucci, Susannah L Shore, Claire Fung, Alison Waghorn, Radu Mihai, Sabapathy Balasubramanian, Arslan Pannu, Shuichi Tatarano, David Velázquez-Fernández, Julie A Miller, Hazel Serrao-Brown, Yufei Chen, Marco Stefano Demarchi, Reza Djafarrian, Helen Doran, Michael J Stechman, Helen Perry, Johnathan G H Hubbard, Cristina Lamas, Philippa Mercer, Janet MacPherson, Supanut Lumbiganon, María Calatayud, Felicia Alexandra Hanzu, Oscar Vidal, Marta Araujo-Castro, César Minguez Ojeda, Theodosios Papavramidis, Pablo Rodríguez de Vera Gómez, Abdulaziz Aldrees, Tariq Altwjry, Nuria Valdés, Cristina Álvarez-Escola, Iñigo García Sanz, Concepción Blanco Carrera, Laura Manjón-Miguélez, María Paz De Miguel Novoa, Mónica Recasens, Rogelio García-Centeno, Cristina Robles Lázaro, Klaas Van Den Heede, Sam Van Slycke, Theodora Michalopoulou, Sebastian Aspinall, Ross Melvin, Joel Wen Liang Lau, Wei Keat Cheah, Man Hon Tang, Han Boon Oh, John Ayuk, Alessandro Parente, Robert P Sutcliffe, on behalf of the International Pheo Study Group

**Affiliations:** ^1^ Department of Surgery, Division of General Surgery, University of Alberta, Faculty of Medicine & Dentistry, Edmonton, Alberta, Canada; ^2^ Department of Urology, The First Affiliated Hospital of Jilin University, Changchun, Jilin, China; ^3^ Department of Urology, Renji Hospital, School of Medicine, Shanghai Jiaotong University, Shanghai, China; ^4^ Division of General, Endocrine and Transplant Surgery, Medical University of Gdańsk, Gdańsk, Poland; ^5^ UOC Chirurgia Endocrina e Metabolica, Fondazione Policlinico Universitario Agostino Gemelli IRCCS, Centro di Ricerca in Chirurgia Endocrina e dell’Obesità, Università Cattolica del Sacro Cuore, Rome, Italy; ^6^ Endocrine Surgical Unit, Royal North Shore Hospital, Northern Sydney Local Health District, Sydney, New South Wales, Australia; ^7^ Department of Endocrine Surgery, Hammersmith Hospital, London, United Kingdom; ^8^ Department of Surgery, Asan Medical Center, University of Ulsan College of Medicine, Seoul, Republic of Korea; ^9^ Department of Endocrine Surgery, Royal Victoria Hospital, Belfast, United Kingdom; ^10^ Endocrine Surgery Unit, Department of Surgery, Oncology and Gastroenterology, University of Padua, Padua, Italy; ^11^ Department of Surgery, London Health Sciences Centre-Victoria Hospital, Western University, London, Ontario, Canada; ^12^ Department of Surgery, Addenbrooke’s Hospital, Cambridge University Hospitals NHS Foundation Trust, Cambridge, United Kingdom; ^13^ Department of Surgery, Lithuanian University of Health Sciences, Kaunas, Lithuania; ^14^ Ozel Saglik Hospital, Centre of Endocrine Surgery, Izmir, Türkiye; ^15^ School of Medicine, Aristoteleio University of Thessaloniki, Thessaloniki, Greece; ^16^ Ege University Hospital, Department of General Surgery, Division of Endocrine Surgery, Izmir, Türkiye; ^17^ Department of Surgery, Amsterdam UMC, University of Amsterdam, Cancer Center Amsterdam, Amsterdam, The Netherlands; ^18^ Endocrine Surgery Unit, University Hospital of Pisa, Pisa, Italy; ^19^ Department of Endocrine and Breast Surgery, Royal Liverpool and Broadgreen University Hospitals Trust, Liverpool, United Kingdom; ^20^ Department of Endocrine Surgery, Churchill Cancer Centre, Oxford University Hospitals NHS Foundation Trust, Oxford, United Kingdom; ^21^ Department of General Surgery, Sheffield Teaching Hospitals Foundation Trust, Sheffield, United Kingdom; ^22^ Department of Urology, Graduate School of Medical and Dental Sciences, Kagoshima University, Kagoshima, Japan; ^23^ Servicio de Cirugía Endocrina y Laparoscopia Avanzada, Departamento de Cirugía, Instituto Nacional de Ciencias Médicas y Nutrición Salvador Zubirán, Mexico City, Mexico; ^24^ Endocrine Surgery Unit, The Royal Melbourne Hospital, Melbourne, Victoria, Australia; ^25^ Cedars-Sinai Medical Center, Department of Surgery, Los Angeles, California, USA; ^26^ Department of Thoracic and Endocrine Surgery and Faculty of Medicine, University Hospitals of Geneva, Geneva, Switzerland; ^27^ Department of Endocrine Surgery, Salford Royal Hospital, Salford, United Kingdom; ^28^ Department of Endocrine Surgery, University Hospital Wales, Cardiff, United Kingdom; ^29^ Department of Endocrine Surgery, St Thomas’ Hospital, London, United Kingdom; ^30^ Endocrinology & Nutrition Department, Complejo Hospitalario Universitario de Albacete, Albacete, Spain; ^31^ Endocrine Surgical Unit, Department of General Surgery, Christchurch Hospital, Christchurch, New Zealand; ^32^ Department of Anaesthesia, Christchurch Hospital, Christchurch, New Zealand; ^33^ Department of Surgery, Division of Urology, Khon Kaen University, Khon Kaen, Thailand; ^34^ Endocrinology & Nutrition Department, Hospital Universitario 12 de Octubre, Madrid, Spain; ^35^ Endocrinology Hospital Clinic University Barcelona, Barcelona, Spain; ^36^ Department of Endocrine Surgery, Hospital Clinic Barcelona, Universitat de Barcelona, IDIBAPS, Barcelona, Spain; ^37^ Department of Endocrinology & Nutrition, Hospital Universitario Ramón y Cajal Madrid, Madrid, Spain; ^38^ Urology Department, Hospital Universitario Ramón y Cajal, Madrid, Spain; ^39^ 1st Propedeutic Department of Surgery, AHEPA University Hospital, Aristotle University of Thessaloniki, Thessaloniki, Greece; ^40^ Endocrinology & Nutrition Department, Hospital Universitario Virgen de la Macarena, Sevilla, Spain; ^41^ King Abdulaziz Medical City, Riyadh, Saudi Arabia; ^42^ Department of Endocrinology and Nutrition, Hospital Universitario Cruces, Biobizkaia, Bizkaia CIBERDEM/CIBERER, Endo-ERN, Barakaldo, Spain; ^43^ Endocrinology & Nutrition Department, Hospital Universitario La Paz Madrid, Madrid, Spain; ^44^ General & Digestive Surgery Department, Hospital Universitario de La Princesa, Madrid, Spain; ^45^ Endocrinology & Nutrition Department, Hospital Universitario Príncipe de Asturias, Madrid, Spain; ^46^ Endocrinology & Nutrition Department, Hospital Universitario Central de Asturias, Oviedo, Spain; ^47^ Instituto de Investigación Sanitaria del Principado de Asturias (ISPA), Oviedo, Spain; ^48^ Hospital Clínico San Carlos, Madrid, Spain; ^49^ Endocrinology & Nutrition Department, Institut Català de la Salut Girona, Girona, Spain; ^50^ Endocrinology & Nutrition Department, Hospital Universitario Gregorio Marañón, Madrid, Spain; ^51^ Endocrinology & Nutrition Department, Hospital Universitario de Salamanca, Salamanca, Spain; ^52^ General and Endocrine Surgery, AZORG, Aalst, Belgium; ^53^ Department of Endocrinology and Nutrition, Joan XXIII University Hospital, Tarragona, Spain; ^54^ Department of General Surgery, Aberdeen Royal Infirmary, Aberdeen, United Kingdom; ^55^ Division of Breast & Endocrine Surgery, Department of General Surgery, Ng Teng Fong General Hospital (NTFGH), National University Health System (NUHS), Singapore, Singapore; ^56^ Department of Endocrinology, Queen Elizabeth Hospital, Birmingham, United Kingdom; ^57^ Institute of Liver Studies, King’s College Hospital, London, United Kingdom; ^58^ Roger Williams Institute of Liver Studies, Faculty of Life Sciences and Medicine, King’s College London, London, United Kingdom; ^59^ Department of Hepatopancreatobiliary and Liver Transplant Surgery, Queen Elizabeth Hospital, Birmingham, United Kingdom

**Keywords:** pheochromocytoma, age, postoperative complications, adrenalectomy, phenotype

## Abstract

**Objective:**

Pheochromocytoma is increasingly diagnosed at the extremes of age due to the availability of genetic screening in young patients and incidental detection on imaging in older patients. The aim of this study was to assess the effect of age on presentation and outcomes after adrenalectomy for pheochromocytoma.

**Methods:**

A retrospective international multicenter cohort study was performed on patients undergoing adrenalectomy for pheochromocytoma between 2012 and 2022 in 49 centers. Patients were divided into three cohorts: ≤40, 41–59, and ≥60 years old. Univariate and multivariate analyses were used to compare cohorts and assess the impact of age on postoperative outcomes.

**Results:**

Of the 2,301 included patients, 551 were ≤40 years (23.9%), 969 were between 41 and 59 years (42.1%), and 781 were ≥60 years (33.9%). Younger cohorts were more likely to have a genetic syndrome (≤40 years 29.4% vs 41–59 years 11.5% vs ≥60 years 6.2%, *P* < 0.001) and bilateral tumors (10.2 vs 2.3 vs 1.3%, *P* < 0.001). On multivariable analysis, overall complications seemed less frequent in older cohorts (adjusted odds ratio (aOR): 0.64, *P* = 0.012, and aOR: 0.66, *P* = 0.054, for 41–59 and ≥60 years versus ≤40 years), while there were no clear trends in severe complications (Clavien–Dindo grade 3a–5) (aOR: 0.81, *P* = 0.481, and aOR: 0.78, *P* = 0.486, respectively) or comprehensive complication index (beta coefficient: −1.49, *P* = 0.046, and −1.66, *P* = 0.083, respectively).

**Conclusion:**

Age is associated with significant differences in presentation in patients undergoing adrenalectomy for pheochromocytoma, but does not have a clear association with postoperative complications. Pheochromocytoma postoperative morbidity is more likely affected by ‘high-risk’ disease rather than exclusively by age.

## Introduction

Pheochromocytoma is a rare neuroendocrine tumor with an annual incidence of 0.6 per 100,000 population (1) and is cured by adrenalectomy in patients with localized disease. Although the majority of pheochromocytomas are diagnosed in the fifth and sixth decades of life (2), a growing number of patients are being diagnosed at the extremes of age. Pheochromocytomas in patients with genetic mutations are diagnosed at a median age of 25 years, while sporadic pheochromocytomas are increasingly being diagnosed incidentally on cross-sectional imaging in older patients (3, 4, 5). There are limited data to suggest that age may influence tumor phenotype; for example, symptomatic and/or multifocal pheochromocytomas are more likely to occur in younger patients (6). Data from small, single-center studies have suggested that older patients have an increased risk of postoperative complications and longer hospitalization (7), while long-term outcomes are unrelated to age (8). Despite the potential relationship between age and phenotype, due to the rarity of pheochromocytoma, few studies have evaluated the effect of age on either presentation or postoperative outcomes.

The aim of this study was to evaluate the effect of age on presentation and postoperative outcomes after adrenalectomy for pheochromocytoma using a multicenter, international cohort.

## Methods

### Study design

A retrospective international, multicenter cohort study was performed. Patients were included from 49 centers in 21 countries. Patients undergoing surgery for pheochromocytoma between January 2012 and December 2022 were included with data collection allowing for a minimum 1-year patient follow-up. Details of this cohort database and study population have been described previously (9, 10).

### Study population and outcomes collected

Pheochromocytoma was confirmed or suspected based on imaging (computed tomography or magnetic resonance imaging) and elevated catecholamine/metanephrine levels in plasma or urine according to individual center protocols. Preoperative factors such as the American Society of Anesthesiologists (ASA) classification (11) were collected. Relevant disease factors collected include indication for surgery, tumor size, association with genetic predisposition, location of tumor, presence of metastases, and undergoing alpha or beta-blockade. Preoperative factors specific to pheochromocytoma were collected, including the timing of alpha blockade cessation, preoperative mean arterial pressure, preoperative admission for intravenous fluids, and metanephrine data, including preoperative 24-h urine, initial presenting, and final preoperative data. Data about surgical approach were collected including incision approach, open versus minimally invasive techniques and if there was a planned nephrectomy.

Multiple postoperative and oncologic outcomes were collected including the need for conversion to open and reason for conversion, postoperative destination, operating time, blood loss, need for transfusion, postoperative hypotension, length of stay (LOS), and resection margin. Composite assessments of complications collected include all complications, including severe complications (Clavien–Dindo (12) grade 3a or greater (both with and without hypotension)), and comprehensive complication index (CCI) scores at 30 days. The CCI score determines the overall burden of complications during postoperative stay, assigning a score from 0 to 100 (13). Long-term outcomes collected include 90-day readmission and mortality and CCI score at 1 year.

### Statistical analysis

Cohorts were created according to the age of diagnosis of the patient: ≤40, 41–59, and ≥60 years old. These age values were used as they align with previous literature assessing the impact of age on outcomes (7, 8), the middle cohort being consistent with average age of diagnosis (2), and <40 aiming to capture the majority of patients with a genetic predisposition (3). This also led to cohorts having similar sample sizes. Srougi *et al.* also used an age threshold of 60 to assess for complications in pheochromocytoma patients (7). We hypothesized that such categories would allow us to better evaluate the impact of age on pheochromocytoma outcomes for patients who may have a higher genetic predisposition (e.g. younger cohorts) versus the impact of frailty (e.g. older cohorts). An additional planned subgroup analysis was run using age cutoffs of ≤65, 66–74, and ≥75, as previous oncology data have suggested significant increases in morbidity in the older cohorts using these cutoffs (14, 15). These age cutoffs were also used by Ma *et al.* and Araujo-Castro *et al.* in their studies (16, 17). A post-hoc analysis was performed using age as a continuous variable in each of the models. Data were presented as mean with standard deviation for continuous variables and as counts and percentages for categorical variables. Median and interquartile ranges were used for metanephrine values due to notable outliers. Continuous variables were compared by regression and categorical variables by chi-squared. Additional analyses comparing ≤40 and 41–59 cohorts and 41–59 and ≥60 cohorts were performed to assess intra-cohort significance. Multivariable logistic regression models were used for assessing the independent effect of variables for bivariate outcomes, while multivariable linear regression models were used for continuous outcomes. Variables were included in the multivariable model if they showed a *P*-value less than 0.1 on univariate analysis or were deemed clinically significant from previous literature or work with this database. Variables with *P*-value less than 0.1 included age, BMI, open versus minimally invasive approach, Charlson comorbidity index, and retroperitoneal versus transabdominal approach; additional variables previously deemed clinically significant include sex, tumor size, planned nephrectomy, and tumor laterality (or bilateral). The independent effects of variables in these models were reported as adjusted odds ratios (aOR) or beta coefficients for logistic and linear models, respectively. Subgroup analyses were performed for each multivariable model with each of the age cohorts. Statistical software inherently assessed for variance inflation factor, with >10 leading to exclusion of the variable from the model. To assess the fit of the models, receiver operating curves (ROC) and Brier score were used for logistic regression models, and *R*^2^ was used for linear regression models. Additional models were created for complications and CCI including only patients of age ≥60 years. For missing data, no imputations or deletions were performed. A complete-case approach was used. *P*-values of less than 0.05 were considered significant. Analysis was performed using STATA17 (STATACorp LP, USA).

### Ethics

Ethics approval was obtained at the lead center (Queen Elizabeth Hospital, Birmingham, United Kingdom; CARMS-18769). Local principal investigators at additional sites were responsible for local ethics approval.

## Results

### Baseline characteristics

A total of 2,301 patients were included, of which 551 were ≤40 years (23.9%), 969 were between 41 and 59 years (42.1%), and 781 were ≥60 years (33.9%, Table 1). The proportion of females was similar among cohorts (44.4–49.0%, *P* = 0.142). Older age cohorts had higher body mass indices (BMI; 26.2 ± 4.8 for ≥60 vs 24.6 ± 4.9 for ≤40, *P* < 0.001), higher Charlson comorbidity indices (3.7 ± 1.7 vs 0.9 ± 1.2, *P* < 0.001), higher ASA scores (score ≥3.0, 45.5 vs 26.7% vs 18.0%), and both hypertension and type 2 diabetes (23.3 vs 6.0%, *P* < 0.001).

**Table 1:** Baseline characteristics of patients undergoing pheochromocytoma resection stratified by age (*n* = 2,301).

	Missing	Age			*P*-value
		≤40 (*n* = 551)	41–59 (*n* = 969)	≥60 (*n* = 781)	
Age (*n* = 2,301)	0 (0.0)	31.0 ± 6.7	50.2 ± 5.4	68.4 ± 6.3	-
BMI (kg/m^2^) (*n* = 1,976)	325 (14.1)	24.6 ± 4.9	25.9 ± 5.0	26.2 ± 4.8	<0.001
Female (*n* = 2,301)	0 (0.0)	270 (49.0)	424 (44.4)	355 (45.5)	0.142
Charlson comorbidity index (*n* = 2,299)	2 (0.1)	0.9 ± 1.2	1.5 ± 1.3	3.7 ± 1.7	<0.001
ASA score (*n* = 2,299)	2 (0.1)				<0.001
1		151 (27.4)	181 (18.7)	52 (6.7)	
2		301 (54.6)	528 (54.6)	373 (47.8)	
3		94 (17.1)	249 (25.7)	324 (41.5)	
4		5 (0.9)	10 (1.0)	31 (4.0)	
Comorbidities (*n* = 1,299)	1,002 (43.5)				<0.001
Type 2 diabetes		18 (3.0)	54 (5.6)	48 (6.1)	
Hypertension		196 (35.6)	347 (35.8)	307 (39.3)	
Both		33 (6.0)	114 (11.8)	182 (23.3)	
Genetic condition (*n* = 2,255)	46 (2.0)	160 (29.4)	109 (11.5)	47 (6.2)	<0.001
Genetic syndrome (*n* = 475)	1,826 (79.4)				<0.001
MEN1		2 (1.1)	1 (0.5)	0 (0.0)	
MEN2A		2 (1.1)	44 (23.7)	15 (14.0)	
MEN2B		17 (9.3)	0 (0.0)	0 (0.0)	
NF-1		19 (10.4)	33 (18.3)	16 (15.0)	
NF-2		1 (0.6)	1 (0.5)	0 (0.0)	
VHL		29 (15.9)	6 (3.2)	2 (1.9)	
Others		24 (13.2)	23 (12.4)	13 (12.2)	
Surgical indication (*n* = 1,886)	415 (18.0)				0.002
Symptomatic/elevated catecholamines		277 (60.0)	482 (59.3)	397 (65.0)	
Suspicious imaging		138 (29.9)	262 (32.2)	148 (24.2)	
Tumor size		34 (7.4)	60 (7.4)	56 (9.2)	
Metastasis		0 (0.0)	2 (0.3)	4 (0.7)	
Others		13 (2.8)	7 (0.9)	6 (1.0)	
Surgical approach (*n* = 2,270)	31 (1.3)				0.237
Open		64 (11.8)	109 (11.4)	93 (12.1)	
Laparoscopic		439 (81.2)	772 (80.7)	634 (82.2)	
Robotic		33 (6.1)	74 (7.7)	42 (5.4)	
Hand-assisted		5 (0.9)	2 (0.2)	3 (0.4)	
Incision approach (*n* = 2,260)	41 (1.8)				<0.001
Transperitoneal		339 (62.8)	587 (61.7)	579 (75.3)	
Retroperitoneal		194 (35.9)	339 (35.7)	183 (23.8)	
Others		7 (1.3)	25 (2.6)	7 (0.9)	
Tumor size (largest tumor cm, *n* = 2,256)	45 (2.0)	4.8 ± 2.6	4.7 ± 2.8	4.7 ± 2.7	0.635
Tumor location (*n* = 2,239)	62 (2.7)				<0.001
Right		250 (47.1)	490 (52.2)	357 (46.4)	
Left		237 (44.6)	429 (45.7)	404 (52.5)	
Bilateral		44 (8.3)	20 (2.1)	8 (1.0)	
Beta blockade (*n* = 2,116)	185 (8.0)	190 (38.4)	282 (31.4)	246 (34.0)	0.031
Alpha blockade (*n* = 2,184)	117 (5.1)	475 (91.9)	847 (92.0)	671 (90.0)	0.395
Alpha blocker used (*n* = 1,919)	382 (16.6)				0.012
Carvedilol		5 (1.1)	9 (1.1)	4 (0.6)	
Doxazosin		170 (37.8)	311 (38.3)	303 (46.2)	
Phenoxybenzamine		245 (54.4)	456 (56.1)	309 (47.1)	
Prazosin		26 (5.8)	24 (3.0)	29 (4.4)	
Terazosin		4 (0.9)	9 (1.1)	7 (1.1)	
Combination/others*		0 (0.0)	4 (0.5)	4 (0.6)	
Alpha blockade discontinuation (*n* = 2,301)	0 (0.0)				<0.001
Day of surgery		232 (42.1)	452 (46.7)	313 (35.9)	
1–10 days prior		29 (5.3)	31 (4.2)	46 (5.3)	
>10 days prior		290 (52.6)	476 (49.1)	512 (58.8)	
Mean arterial pressure (mmHg, *n* = 2,087)	214 (9.3)	99.4 ± 16.8	100.5 ± 20.0	98.7 ± 18.4	0.367
Plasma metanephrine initial (median ± IQR, *n* = 835)	1,466 (63.7)	4,280.8 ± 8,889	3,723.5 ± 6,161.7	2,961 + 6,066	0.180
Plasma metanephrine preoperative (*n* = 774)	1,527 (66.3)	609 ± 2,500	856 ± 2,536.1	1,046.9 ± 2,260	0.210
24-h urine metanephrine (*n* = 861)	1,440 (62.6)	11.0 ± 31.0	8.8 ± 24.2	6.6 ± 26.0	0.110
Admitted preoperatively for IV fluids (*n* = 2,143)	158 (6.9)	186 (36.0)	293 (32.4)	256 (35.5)	0.357
Planned nephrectomy (*n* = 1,933)	368 (16.0)	3 (0.6)	13 (1.6)	7 (1.1)	0.316
Extra-adrenal metastases (*n* = 2,183)	118 (5.1)	9 (1.7)	16 (1.8)	14 (1.9)	0.752

Values presented as mean ± SD for continuous variables and *n* (%) for categorical variables.

ASA, American Society of Anesthesiology; BMI, body mass index.

* This included use of prazosin and phenoxybenzamine (*n* = 1), verapamil (*n* = 4), doxazosin and alfuzosin (*n* = 2), doxazosin and tamsulosin (*n* = 1), and nicardipine (*n* = 1).

### Differences in presentation and phenotype per age cohort

Younger patients were more likely to have a genetic component (≤40 years 29.4% vs ≥60 years 6.2%, *P* < 0.001). Genetic syndromes were also different among the age cohorts (*P* < 0.001), with major differences secondary to increased presence of MEN2B (9.3 vs 0.0% for both other cohorts) and VHL (15.9 vs 1.9–3.2% in other cohorts) in ≤40 and increase in MEN2A in older cohorts (14.0% in ≥60 vs 23.7% in 41–59 vs 1.1% in ≤40). Though patients were most likely to have surgery due to being symptomatic or having elevated catecholamines (59.3–65.0%), older cohorts were more likely to have an operation due to tumor size (≤40 years and 41–59 years 7.4% vs ≥60 years 9.2%). Younger cohorts were instead more likely to have an operation due to suspicious imaging (≤40 years 29.9% vs 41–59 years 32.2% and ≥60 years 24.2%). The youngest cohort was most likely to have bilateral tumors (10.2%) compared to the two older cohorts (1.3 and 2.3%, *P* < 0.001). Tumor size was not significantly different among groups (*P* = 0.635). There was no difference in the proportion of patients with extra-adrenal metastases (1.7–1.9%, *P* = 0.752) or the percentage of patients with planned nephrectomy (0.6–1.6%, *P* = 0.316). During intergroup comparison, surgical indication and incision approach were not significant between ≤40 and 41–59 cohorts but remained significant between 41 and 59 and ≥60 cohorts.

### Comparison of operative approaches by age

Regarding surgical approach, there was no difference in minimally invasive vs open techniques between cohorts, with most undergoing a minimally invasive approach. The technical approach, however, was significantly different among cohorts with patients ≥60 being more likely to undergo a transperitoneal approach (75.3%) than those in 41–59 (61.7%) and ≤40 (62.8%, *P* < 0.001) cohorts, and those older less likely to undergo a retroperitoneal approach (23.8% in ≥60 vs 35.7–35.9% in other cohorts).

### Outcome comparison stratified by age

Operating time was similar among cohorts in univariate analysis, but this did not take surgical or incision approach into account (126.6–136.3 min, *P* = 0.076, Table 2). Estimated blood loss was also not significantly different (155.5–184.4 mL, *P* = 0.872). Postoperatively, the ≥60 cohort was more likely to require going to high dependency unit (HDU; 19.5 vs 16.6% and 2.5% for 41–59 and ≤40, respectively), while the 41–59 cohort was most likely to go to ward (49.0 vs 45.4% and 40.6% for ≤40 and ≥60) and the ≤40 cohort was the most likely to go to ICU (39.3 vs 32.4% for 41–59 and 37.4% for ≥60).

**Table 2:** Postoperative outcomes of patients undergoing resection for pheochromocytoma stratified by age.

	Missing	Age			*P*-value
		≤40 (*n* = 551)	41–59 (*n* = 969)	≥60 (*n* = 781)	
Postoperative location (*n* = 2,253)	48 (2.1)				0.001
Ward		242 (45.4)	465 (49.0)	312 (40.6)	
ICU		210 (39.3)	308 (32.4)	287 (37.4)	
High acuity unit		67 (12.5)	158 (16.6)	150 (19.5)	
Conversion to open (*n* = 2,301)	0 (0.0)	15 (2.7)	34 (3.5)	28 (3.2)	0.706
Reason for conversion (*n* = 2,301)	0 (0.0)				0.394
Bleeding		26 (4.7)	32 (3.3)	44 (5.1)	
Instability		2 (0.4)	4 (0.4)	3 (0.3)	
Tumor size		1 (0.2)	5 (0.5)	5 (0.6)	
Others		2 (0.4)	13 (1.3)	9 (1.0)	
Operative time (min, *n* = 1,854)	447 (19.4)	136.3 ± 76.6	126.6 ± 78.9	127.4 ± 69.3	0.076
Estimated blood loss (mL, *n* = 1,571)	730 (31.7)	159.7 ± 462.6	184.4 ± 909.4	155.5 ± 516.3	0.872
Transfusion required (*n* = 2,206)	95 (4.1)	28 (5.3)	55 (5.9)	52 (7.0)	0.488
Length of stay (days, *n* = 2,233)	68 (3.0)	5.7 ± 4.6	5.6 ± 4.3	5.3 ± 4.3	0.041
Complication during stay (*n* = 2,192)	109 (4.7)	110 (20.3)	171 (17.9)	174 (22.5)	0.057
Complication CDC 3a or greater during stay (*n* = 2,275)	26 (1.1)	36 (6.6)	55 (5.7)	55 (7.1)	0.477
Complication CDC 3a or greater, excluding hypotension (*n* = 1,990)	311 (13.5)	9 (1.9)	14 (1.6)	14 (2.1)	0.804
Postoperative hypotension					
<24 h (*n* = 2,192)	109 (4.7)	122 (23.4)	187 (20.2)	159 (21.4)	0.356
>24 h (*n* = 1,858)	443 (19.3)	33 (7.5)	47 (6.1)	47 (7.3)	0.553
Resection margin (*n* = 1,837)	464 (20.2)				0.447
R0		419 (95.0)	756 (94.9)	559 (93.3)	
R1		19 (4.3)	39 (4.9)	35 (5.8)	
R2		3 (0.7)	2 (0.3)	5 (0.8)	
CCI					
At discharge (*n* = 2,292)	9 (0.4)	5.8 ± 13.3	5.1 ± 11.9	6.5 ± 14.2	0.203
At one year (*n* = 2,293)	8 (0.3)	6.4 ± 14.1	5.4 ± 12.2	7.1 ± 14.9	0.223
Readmission within 90 days (*n* = 2,254)	47 (2.0)	12 (2.2)	7 (0.7)	18 (2.4)	0.015
Mortality within 90 days (*n* = 2,301)	0 (0.0)	1 (0.2)	1 (0.1)	2 (0.2)	0.799

Values presented as mean ± SD for continuous variables and *n* (%) for categorical variables.

CCI, comprehensive complication index; CDC, Clavien–Dindo classification; ICU, intensive care unit.

Younger patients had the longest LOS (5.7 days vs 5.6 and 5.3 days for 41–59 and ≥60-year cohorts, *P* = 0.041). Postoperative hypotension was similar among cohorts. The oldest cohort had a higher, though non-statistically significant, rate of severe complications (22.5%) compared with the 41–59 (17.9%) and ≤40 (20.3%) cohorts, with the *P*-value approaching significance (*P* = 0.057). Rates of severe complications (CDC 3a or greater) were similar among cohorts (5.7–7.1%; *P* = 0.477), including severe complications excluding postoperative hypotension as a complication (1.6–2.1%, *P* = 0.804).

Resection margins were similar among cohorts with 93.3–95.0% achieving R0 margins. CCI scores at discharge (5.1–6.5) and 1 year (5.4–7.1) were not statistically significant among groups. Readmission at 90 days was significantly different with the youngest cohort (2.2%) and oldest cohort (2.4%) having higher rates than those in the 41–59 cohort (0.7%, *P* = 0.015). Mortality within 90 days from complications was similar among groups (*P* = 0.799), with a rate of 0.1% (*n* = 1) for the 41–59 cohort and a rate of 0.2% for the ≤40 (*n* = 1) and ≥60 (*n* = 2) cohorts.

During intergroup comparison, notable differences include that operative time was significantly different between the ≤40 and 41–59 cohorts (*P* = 0.035) but length of stay was not significantly different for either the ≤40 vs 41–59 or 41–59 vs ≥60 comparisons. All complications (*P* = 0.017), CCI at discharge (*P* = 0.020), and CCI at 1 year (*P* = 0.008) were also significantly higher in the ≥60 vs 41–59 cohort.

### Multivariable models assessing factors associated with postoperative complications

The first model assessed factors associated with any postoperative complication (Table 3). Older cohorts were less likely to experience any complication (versus ≤40, 41–59 years aOR: 0.64, *P* = 0.012, and ≥60 years aOR: 0.66, *P* = 0.054). Minimally invasive surgery (versus open, laparoscopy aOR: 0.34, *P* < 0.001, and robotic aOR: 0.43, *P* = 0.006) was associated with lower complication rates. Higher Charlson comorbidity index (aOR: 1.19, *P* < 0.001) and tumor size (per cm, aOR: 1.14, *P* < 0.001) were associated with significantly higher complication rates. The ROC area under the curve is 0.701 and the Brier score is 0.1346, indicating a good fit.

**Table 3:** Multivariate logistic regression model for predictors of complication following resection for pheochromocytoma. The sample size for the model is 1,627.

	aOR	95% CI	*P*-value
Age (versus ≤40)			
41–59	0.64	0.45–0.91	0.012
≥60	0.66	0.44–1.01	0.054
BMI (per kg/m^2^)	1.00	0.97–1.03	0.989
Surgical approach (versus open)			
Laparoscopic	0.34	0.23–0.50	<0.001
Robotic	0.43	0.24–0.79	0.006
Hand-assisted	0.41	0.04–4.66	0.474
Male sex (versus female)	0.99	0.75–1.29	0.920
Charlson comorbidity index	1.19	1.10–1.29	<0.001
Tumor size (largest, cm)	1.14	1.08–1.201	<0.001
Tumor laterality (versus right-sided)			
Left-sided	0.98	0.74–1.29	0.882
Bilateral tumor	1.32	0.67–2.58	0.424
Planned nephrectomy	0.98	0.37–3.24	0.860
Retroperitoneal approach (versus transperitoneal)	1.10	0.74–1.30	0.901

The area under the curve for the receiver operating curve is 0.701, and the Brier score is 0.1346.

Another multivariable logistic regression model was generated to assess for variables associated with severe complications, classified as Clavien–Dindo grade 3a or greater complications (which excluded intraoperative complications; ROC area under the curve is 0.661, Brier score is 0.1582, Table 4). Age was not associated with likelihood of severe complications.

**Table 4:** Multivariate logistic regression model for predictors of complication classified as Clavien–Dindo grade 3a or greater following resection for pheochromocytoma. The sample size for the model is 1,609.

	aOR	95% CI	*P*-value
Age (versus ≤40)			
41–59	0.81	0.44–1.47	0.481
≥60	0.78	0.38–1.58	0.486
BMI (per kg/m^2^)	1.04	0.99–1.08	0.120
Surgical approach (versus open)			
Laparoscopic	0.39	0.21–0.69	0.001
Robotic	0.17	0.04–0.74	0.018
Hand-assisted	1.95	0.17–22.48	0.593
Male sex (versus female)	1.36	0.85–2.17	0.198
Charlson comorbidity index	1.09	0.95–1.25	0.220
Tumor size (largest, cm)	1.10	1.02–1.19	0.010
Tumor laterality (versus right-sided)			
Left-sided	1.38	0.86–2.21	0.183
Bilateral tumor	1.93	0.68–5.43	0.215
Planned nephrectomy	0.88	0.19–3.98	0.864
Retroperitoneal approach (versus transperitoneal)	0.64	0.37–1.11	0.109

The area under the curve for the receiver operating curve is 0.661, and the Brier score is 0.1582.

The third model created assessed factors associated with CCI scores at discharge (*R*^2^ is 0.1118, Table 5). Patients in the 41–59-year cohort were more likely to have a lower score (−1.49, −3.01–0.02) while there was a non-significant trend in the ≥60 cohort (−1.66, −3.54–0.22).

**Table 5:** Multivariate linear regression model for predictors of total comprehensive complication index at discharge following resection for pheochromocytoma. The sample size for the model is 1,626.

	Beta coefficient	95% CI	*P*-value
Age (versus ≤40)			
41–59	−1.49	−3.01–0.02	0.046
≥60	−1.66	−3.54–0.22	0.083
BMI (per kg/m^2^)	0.07	−0.06–0.19	0.296
Surgical approach (versus open)			
Laparoscopic	−8.20	−10.25–−6.15	<0.001
Robotic	−7.83	−10.70–−4.96	<0.001
Hand assisted	−2.45	−14.26–9.36	0.648
Male sex (versus female)	0.13	−1.03–1.30	0.822
Charlson comorbidity index	0.67	0.29–1.05	0.001
Tumor size (largest, cm)	0.66	0.41–0.91	<0.001
Tumor laterality (versus right-sided)			
Left-sided	0.07	−1.12–1.25	0.914
Bilateral tumor	1.47	−1.69–4.64	0.362
Planned nephrectomy	3.56	−2.28–9.41	0.232
Retroperitoneal approach (versus transperitoneal)	−0.29	−1.52–0.93	0.640

The value for *R*^2^ for the model is 0.1118.

### Multivariable models to assess factors associated with postoperative risk for each age cohort

Subgroup analyses were run for each age cohort individually for all complications, serious complications and CCI to determine if any preoperative factors uniquely affected postoperative outcomes of each of these cohorts (Supplementary Tables 1, 2, 3 (see section on Supplementary materials given at the end of the article)). Minimally invasive surgery seemed to have a protective effect for all outcomes assessed in most age cohorts. Other factors were less consistently significant, such as Charlson comorbidity index, tumor size, tumor laterality, and planned nephrectomy.

### Subgroup analysis using age cohorts of ≤65, 66–74, and ≥75

Additional multivariable analyses using age cohorts of ≤65, 66–74, and ≥75 did not find an association of age with any complication, including severe complications, or CCI (Supplementary Table 4).

### Subgroup analysis using age as a continuous variable

Furthermore, we performed multivariate analyses using age as a continuous variable and did not find an association of age with any complication, including severe complications, or CCI (Supplementary Table 5).

## Discussion

This is the largest study to date that has evaluated the effect of age on both presentation and postoperative outcomes of patients undergoing adrenalectomy for pheochromocytoma. Our main finding is that age is significantly associated with differences in disease phenotype but not necessarily with postoperative complications, with no clear association of age with many indices of complication severity. As expected, younger patients were significantly more likely to have hormonally active (symptomatic) tumors with an association with genetic syndromes. Though there were differences in rates of postoperative complications on bivariate analysis, such as postoperative ICU stay and longer LOS, age was not independently associated with severe complications.

The association between young age and hormonally active tumors corroborates existing data derived from several small studies (3, 4, 6), and may explain why younger cohorts had increased need for ICU support and longer LOS on bivariate analysis. There may be a more significant hemodynamic effect when catecholamine-releasing tumors are removed in younger patients; our data show a non-statistically significant increased rate of postoperative hypotension (for both <24 h and >24 h) to be highest in the ≤40 cohort. The difference in outcomes is also likely to be influenced by patient selection, since a higher proportion of older patients with significant comorbidity may be considered unfit for adrenalectomy, potentially skewing the postoperative outcomes, while younger patients with similarly aggressive tumors may be considered fit for surgery. This highlights the importance of careful patient selection combined with optimal perioperative management to minimize the risk of postoperative complications and mortality. Importantly, all centers that contributed data to this international cohort study were identified as high-volume centers, and therefore, the results of this study may not be generalizable to small-volume centers (18).

The second important outcome from this study is that although differences in complications exist, after accounting for important disease factors such as planned nephrectomy, baseline comorbidity, and surgical approach, serious complications and CCI at discharge were similar between cohorts; this likely also explains the difference in unadjusted results and results adjusted for the above factors. The lack of association was also present when cohorts were divided into ≤65, >65 to <75, and ≥75 or when age was used as a continuous variable, signifying that age only partially contributes to postoperative risk in this heterogeneous disease. These findings parallel a previous study from Ma *et al.* that noted no association of age ≥45 with postoperative complications (CDC ≥2) (17). Similarly, Bai *et al.* also showed no association of age with postoperative complications (Clavien–Dindo grade ≥ 2) on multivariable analysis when assessed as a continuous variable (19). Conversely, other existing studies in the literature have noted a possible signal for increased complications in patients with increasing age, including Srougi *et al.* who noted in their single-center analysis of 46 patients that older patients (>60) were three times more likely to have a clinical complication on univariate analysis (7). A multicenter retrospective cohort study (PHEO-RISK) also noted age ≥65 to be associated with intraoperative or postoperative complications (Clavien–Dindo grade ≥ 2) (16). Though certain analyses in this study showed an association of younger age with all postoperative complications, this is less likely secondary to physiologic factors.

Utilizing age for risk stratification should take into consideration tumor phenotype and genetic predisposition which may play a more significant role. Aggarwal *et al.* suggested age likely plays a role in tumor biology and resultant clinical, radiological, and tumor characteristics, after noting similar significant differences in presentation depending on age of diagnosis to our data (5). There is a complex interplay as these factors, such as age, tumor phenotype, and genetic predisposition, are inter-related, and there are limited data on ‘high-risk’ tumors in patients without a genetic mutation. Other potentially modifiable factors associated with risk of complications included an open procedure, which may be due to selection bias as open techniques are recommended for large or invading tumors (20). Charlson comorbidity index was also significant in some analyses, and thus, control of underlying comorbidities may be helpful. To note, while the logistic regression models reported reasonable AUC, the *R*^2^ for the linear regression model for CCI was low, which suggests that our model does not fully capture pertinent factors relating to postoperative CCI. Non-modifiable factors include tumor size, for which center and surgeon expertise will likely dictate approach; utilization of minimally invasive strategies where possible may help circumvent some of this risk. Preoperative planning for pheochromocytomas is unique with multiple decisions, including incision and use of minimally invasive surgery, and stratification of disease into higher or lower risk can help to optimize patient-centered procedures and postoperative management. Given the significance of positive genetic testing, for future generations as well as its role in tumor phenotype, routine testing for pheochromocytomas is recommended regardless of age, as encouraged by published guidelines (20).

This study has some limitations. Due to the significant diversity in population, there may be heterogeneity in practice patterns among centers, which may limit interpretation of certain results (e.g. postoperative destination, LOS). Furthermore, given the multicenter nature of this study, surgeons with higher volumes of pheochromocytoma resection may have a high proportion of their cases represented, thus limiting generalizability to lower resource or lower volume centers. There are also likely inter-center differences in practices, as well as differences in practices from high-volume centers to other centers, which can increase bias and limit generalizability further. Although data on many complications were collected, detailed collection of individual complications is limited; thus, it is difficult to determine which are driving the significant results in the multivariate analysis for age. Furthermore, only patients undergoing adrenalectomy for pheochromocytoma were captured by this study; thus, patients with non-resectable disease, disease not treated with surgery, or other indications for adrenalectomy were excluded. Selection bias may also exist as only fit patients of higher age may have been included. Other confounding factors include frailty, which was not collected, or tumor biology, which was not incorporated into the multivariate analysis. There are also missing data for certain variables which could affect results; notably, there remained greater than 1,500 patients for the multivariate analyses performed. The complete-case approach in this case may, however, bias the reported estimates as granular detail of catecholamines and genetic syndromes could impact results: for example, full availability of data may show increased rates of complications in patients with higher catecholamine levels. An additional limitation of this was the inability to characterize catecholamine secretion of different patient cohorts. Due to missing data, meaningful comparison of catecholamine levels could not be performed, which likely play a role in patient presentation and possibly outcomes. Catecholamine data were also limited by a lack of uniform reporting on units and due to significant variance in number of patients that each center reported, center-adjusted outcomes could not be meaningfully described. As there is likely center-to-center variance in postoperative protocols, this may contribute partially to the results seen for ICU destination and LOS. Also, as a complete-case approach was used, this may also introduce bias.

Nonetheless, this study should be interpreted in the context of its strengths. It represents the largest cohort of patients undergoing resection for solely pheochromocytoma using a multicontinental dataset. Results suggest that clinical presentation is often associated with different age cohorts and relates to outcomes more so than age; as such, clinicians should understand differences in pheochromocytoma in various age groups and how it may affect perioperative outcomes. Considering the complexity of these interactions, performing adrenalectomy at high-volume centers remains an important consideration. Future directions include a detailed biochemical analysis of tumor phenotypes based on age.

## Conclusion

Patient cohorts are significantly affected by age of presentation, with key differences in baseline comorbidities and tumor pathology. Differences in both presentation and outcomes may be affected by younger patients representing higher-risk tumor groups. Age was not found to be independently associated with severe complications.

## Supplementary materials



## Declaration of interest

The authors of this manuscript do not have any conflicts of interest that could be perceived as prejudicing the impartiality of the research reported.

## Funding

This research did not receive any specific grant from any funding agency in the public, commercial, or not-for-profit sector.

## Ethics

The study was approved with registration, CARMS-18769.
