# Supplementary material for: Evaluation of differences in presentation and postoperative outcomes after adrenalectomy for pheochromocytoma according to age
Source: Endocr Connect. 2026 Jul 28;15(7):e250604. doi: 10.1530/EC-25-0604 (PMC13427973; doi:10.1530/EC-25-0604)
Supplement: Supplementary file 1 [file EC-25-0604_supplementary_tables.pdf]

Supplementary Table 1. Multivariate logistic regression model for predictors of complication following resection for pheochromocytoma, stratified by cohort.

	≤40-year cohort[a]			41-59-year cohort[b]			≥60-year cohort[c]		
	aOR	95% CI	p-value	aOR	95% CI	p-value	aOR	95% CI	p-value
BMI (per kg/m^2)	1.01	0.96 – 1.07	0.644	0.98	0.93 – 1.03	0.365	1.01	0.96-1.06	0.677
Surgical approach (versus open)									
Laparoscopic	0.278	0.13 – 0.57	0.001	0.58	0.28 – 1.20	0.144	0.26	0.14-0.49	<0.001
Robotic	0.22	0.06 – 0.77	0.017	0.89	0.34 – 2.33	0.806	0.32	0.11-0.97	0.044
Hand-assisted	1.10	0.06 – 19.94	0.948	1.00	-	-	*	-	-
Male sex (versus female)	0.80	0.47 – 1.35	0.398	1.19	0.74 – 1.90	0.468	1.04	0.66-1.65	0.856
Charlson comorbidity index	1.06	0.88 – 1.28	0.559	1.19	1.03 – 1.39	0.020	1.26	1.12-1.43	<0.001
Tumor size (largest, cm)	1.00	0.90 – 1.11	0.986	1.27	1.16 – 1.39	<0.001	1.16	1.05-1.28	0.002
Tumor laterality (versus right-sided)									
Left-sided	0.99	0.58 – 1.70	0.968	1.27	0.80 – 2.03	0.311	0.78	0.48-1.24	0.289
Bilateral tumor	0.99	0.40 – 2.48	0.985	2.92	0.87 – 9.84	0.084	2.80	0.54-14.5	0.221
Planned nephrectomy	2.61	0.18 – 37.39	0.480	0.24	0.04 – 1.46	0.121	0.60	0.31-1.16	0.126
Retroperitoneal approach (versus transperitoneal)	1.17	0.69 – 1.99	0.552	0.86	0.54 – 1.36	0.508	4.39	1.65-11.7	0.003

*Predicted outcome perfectly so removed from analysis

[a]The area under the curve for the receiver-operating curve is 0.613 and the Brier score is 0.1396. Sample size for model is 399.

[b]The area under the curve for the receiver-operating curve is 0.671 and the Brier score is 0.1384. Sample size for model is 670.

[c]The area under the curve for the receiver-operating curve is 0.681 and the Brier score is 0.1389. Sample size for model is 556.

Supplementary Table 2. Multivariate logistic regression model for predictors of complication classified as Clavien-Dindo grade 3a or greater following resection for pheochromocytoma, stratified by cohort.

	≤40-year cohort[a]			41-59-year cohort[b]			≥60-year cohort[c]		
	aOR	95% CI	p-value	aOR	95% CI	p-value	aOR	95% CI	p-value
BMI (per kg/m^2)	1.06	0.98-1.16	0.162	0.97	0.89-1.06	0.482	1.08	1.00-1.17	0.042
Surgical approach (versus open)									
Laparoscopic	0.65	0.19-2.23	0.494	0.74	0.22-2.48	0.630	0.23	0.09-0.58	0.002
Robotic	*	-	-	0.61	0.10-3.76	0.594	*	-	-
Hand-assisted	5.83	0.24-143.81	0.281	*	-	-	*	-	-
Male sex (versus female)	0.94	0.37-2.40	0.894	1.56	0.69-3.51	0.284	1.61	0.72-3.62	0.245
Charlson comorbidity index	1.21	0.90-1.61	0.202	1.21	0.96-1.54	0.116	0.95	0.77-1.17	0.636
Tumor size (largest, cm)	1.02	0.88-1.18	0.795	1.22	1.07-1.39	0.002	1.11	0.96-1.28	0.154
Tumor laterality (versus right-sided)									
Left-sided	0.98	0.37-2.62	0.974	2.63	1.13-6.12	0.024	1.09	0.50-2.36	0.828
Bilateral tumor	1.70	0.41-7.10	0.465	7.20	1.34-38.75	0.021	*	-	-
Planned nephrectomy	5.02	0.22-112.07	0.310	0.30	0.02-4.54	0.385	1.76	0.16-19.19	0.644
Retroperitoneal approach (versus transperitoneal)	0.60	0.19-1.88	0.383	0.83	0.38-1.80	0.629	0.33	0.08-1.34	0.121

* Predicted outcome perfectly so removed from analysis

[a]The area under the curve for the receiver-operating curve is 0.621 and the Brier score is 0.1544. Sample size for model is 365.

[b]The area under the curve for the receiver-operating curve is 0.638 and the Brier score is 0.1575. Sample size for model is 663.

[c]The area under the curve for the receiver-operating curve is 0.644 and the Brier score is 0.1538. Sample size for model is 517.

Supplementary Table 3. Multivariate linear regression model for predictors of total Comprehensive Complication Index at discharge following resection for pheochromocytoma, stratified by cohort.

	≤40-year cohort[a]			41-59-year cohort[b]			≥60-year cohort[c]		
	Beta coefficient	95% CI	p-value	Beta coefficient	95% CI	p-value	Beta coefficient	95% CI	p-value
BMI (per kg/m^2)	0.15	-0.123 to 0.43	0.291	-0.07	-0.25 to 0.10	0.400	0.18	-0.05 to 0.41	0.116
Surgical approach (versus open)									
Laparoscopic	-8.72	-13.01 to 4.42	<0.001	-3.39	-6.44 to -0.33	0.030	-12.22	-15.78 to -8.66	<0.001
Robotic	-9.40	-15.58 to 3.22	0.003	-2.89	-6.83 to1.05	0.150	-11.22	-16.77 to -5.66	<0.001
Hand-assisted	10.09	-8.28 to 28.47	0.281	-5.45	-26.48 to 15.57	0.611	-35.64	-62.39 to -8.90	0.009
Male sex (versus female)	-0.65	-3.22 to1.92	0.620	0.98	-0.65 to 2.62	0.238	-0.08	-2.17 to 2.00	0.938
Charlson comorbidity index	0.68	-0.31 to 1.66	0.176	0.54	-0.04 to 1.11	0.063	0.75	0.16 to 1.33	0.012
Tumor size (largest, cm)	0.02	-0.53 to 0.56	0.953	1.16	0.82 to 1.51	<0.001	0.64	0.17 to 1.11	0.007
Tumor laterality (versus right-sided)									
Left-sided	0.14	-2.55 to 2.83	0.920	1.11	-0.53 to 2.73	0.184	-0.87	-2.96 to 1.23	0.416
Bilateral tumor	0.878	-3.72 to 5.48	0.708	4.80	-0.39 to 10.00	0.070	3.11	-7.87 to 14.09	0.578
Planned nephrectomy	16.76	0.92 to 32.60	0.038	-2.89	-10.50 to 4.73	0.457	12.22	1.17 to 23.26	0.030
Retroperitoneal approach (versus transperitoneal)	0.05	-2.61 to 2.70	0.971	-0.30	-1.93 to 1.34	0.723	-0.51	-2.88 to 1.85	0.671

* Predicted outcome perfectly so removed from analysis

[a]The value for R^2 for the model is 0.0937. Sample size for model is 398.

[b]The value for R^2 for the model is 0.1164. Sample size for model is 671.

[c]The value for R^2 for the model is 0.1791. Sample size for model is 557.

Supplementary Table 4. Multivariate logistic regression model for predictors of complication following resection for pheochromocytoma, stratified by age cohorts of ≤65, >65 to <75 and ≥75.

	Any complication cohort[a]			Complication grade ≥3a[b]			CCI[c]		
	aOR	95% CI	p-value	aOR	95% CI	p-value	Beta coefficient	95% CI	p-value
Age (vs ≤65)									
>65 to <75	0.93	0.61-1.39	0.711	0.76	0.36-1.61	0.471	-0.09	-1.95 to 1.78	0.928
≥75	1.09	0.63-1.88	0.771	1.87	0.82-4.26	0.134	0.77	-1.85 to 3.39	0.564
BMI (per kg/m^2)	1.00	0.97-1.02	0.772	1.04	0.99-1.08	0.133	0.05	-0.07 to 0.18	0.416
Surgical approach (versus open)									
Laparoscopic	0.34	0.23-0.49	<0.000	0.37	0.20-0.66	0.001	-8.29	-10.34 to -6.24	<0.001
Robotic	0.42	0.23-0.76	0.004	0.16	0.04-0.72	0.017	-7.96	-10.83 to -5.09	<0.001
Hand-assisted	0.46	0.04-5.32	0.537	1.70	0.14-21.40	0.681	-2.14	-13.97 to 9.68	0.723
Male sex (versus female)	0.97	0.74-1.29	0.901	1.34	0.84-2.15	0.216	0.06	-1.11 to 1.23	0.919
Charlson comorbidity index	1.16	1.07-1.27	<0.001	1.05	0.92-1.20	0.500	0.50	0.14 to 0.87	0.007
Tumor size (largest, cm)	1.14	1.08-1.20	<0.001	1.11	1.03-1.19	0.008	0.66	0.41 to 0.91	<0.001
Tumor laterality (versus right-sided)									
Left-sided	0.98	0.75-1.29	0.900	1.33	0.83-2.13	0.239	0.06	-1.12 to 1.25	0.915
Bilateral tumor	1.53	0.80-2.96	0.201	2.03	0.74-5.60	0.171	2.02	-1.10 to 5.15	0.205
Planned nephrectomy	1.02	0.35-2.98	0.977	0.78	0.17-3.67	0.757	3.29	-2.55 to 9.14	0.269
Retroperitoneal approach (versus transperitoneal)	0.96	0.73-1.28	0.798	0.64	0.37-1.11	0.113	-0.33	-1.56 to 0.89	0.593

[a]The area under the curve for the receiver-operating curve is 0.690 and the Brier score is 0.1348. Sample size for model is 1627.
[b]The area under the curve for the receiver-operating curve is 0.655 and the Brier score is 0.1585. Sample size for model is 1609.
[c]The value for R^2 for the model is 0.1096. Sample size for model is 1626.

Supplementary Table 5. Multivariate logistic regression model for predictors of complication following resection for pheochromocytoma, analyzed with age as a continuous variable.

	Any complication cohort[a]			Complication grade ≥3a[b]			CCI[c]		
	aOR	95% CI	p-value	aOR	95% CI	p-value	Beta coefficient	95% CI	p-value
Age	0.99	0.98-1.00	0.07	1.00	0.98-1.02	0.890	-0.03	-0.08 to 0.016	0.187
BMI (per kg/m^2)	0.99	0.97-1.03	0.915	1.03	0.99-1.08	0.149	0.06	-0.06 to 0.18	0.347
Surgical approach (versus open)									
Laparoscopic	0.34	0.23-0.50	<0.001	0.38	0.21-0.69	0.001	-8.22	-10.27 to -6.17	<0.001
Robotic	0.43	0.24-0.78	0.005	0.16	0.04-0.72	0.017	-7.87	-10.74 to -5.00	<0.001
Hand-assisted	0.46	0.04-5.15	0.532	2.13	0.19-24.44	0.544	-2.09	-13.90 to 9.71	0.728
Male sex (versus female)	0.98	0.75-1.28	0.882	1.35	0.84-2.15	0.210	0.09	-1.07 to 1.26	0.875
Charlson comorbidity index	1.21	1.11-1.31	<0.001	1.06	0.93-1.22	0.397	0.69	0.31 to 1.07	<0.001
Tumor size (largest, cm)	1.14	1.08-1.20	<0.001	1.10	1.02-1.19	0.011	0.66	0.41 to 0.91	<0.001
Tumor laterality (versus right-sided)									
Left-sided	0.99	0.75-1.0	0.950	1.37	0.86-2.20	0.185	0.09	-1.10 to 1.27	0.887
Bilateral tumor	1.35	0.69-2.66	0.382	2.14	0.75-6.04	0.153	1.62	-1.56 to 4.80	0.318
Planned nephrectomy	1.05	0.36-3.11	0.923	0.85	0.19-3.82	0.828	3.45	-2.40 to 9.29	0.248
Retroperitoneal approach (versus transperitoneal)	0.97	0.73-1.29	0.851	0.63	0.36-1.09	0.100	-0.32	-1.55 to 0.91	0.608

[a]The area under the curve for the receiver-operating curve is 0.699 and the Brier score is 0.1348. Sample size for model is 1627.

[b]The area under the curve for the receiver-operating curve is 0.654 and the Brier score is 0.1583. Sample size for model is 1609.
[c]The value for R^2 for the model is 0.1104. Sample size for model is 1626.
